# Epidemiology of artherial hypertension in pregnants

**DOI:** 10.31744/einstein_journal/2020AO4682

**Published:** 2019-10-22

**Authors:** Marilda Gonçalves de Sousa, Reginaldo Guedes Coelho Lopes, Maria Luiza Toledo Leite Ferreira da Rocha, Umberto Gazi Lippi, Edgar de Sousa Costa, Célia Maria Pinheiro dos Santos

**Affiliations:** 1 Instituto de Assistência Médica ao Servidor Público Estadual São PauloSP Brazil Instituto de Assistência Médica ao Servidor Público Estadual, São Paulo, SP, Brazil.; 2 Universidade Federal de São Paulo São PauloSP Brazil Universidade Federal de São Paulo, São Paulo, SP, Brazil.

**Keywords:** Pregnancy complications/epidemiology, Pregnancy-induced hypertension/epidemiology, Hospitals, Health Services

## Abstract

**Objective:**

To investigate the epidemiological data of hypertension in pregnant women, as well as to identify its possible associated events.

**Methods:**

Data collection was performed at the high-risk prenatal outpatient clinic and in the maternity ward at a public hospital in the São Paulo city, during the morning and afternoon periods, from October 2015 to July 2016. A questionnaire with 22 questions prepared by the researchers was used. The margin of error was 5% and the confidence level was 95%. For the calculation, the two-proportion equality, Pearson correlation and ANOVA tests were used.

**Results:**

Among the interviewees, 43% had chronic hypertension, 33.3% presented with up to 20 weeks of gestation, 23.7% presented after the 20th week of gestation, 62.3% were between 18 and 35 years of age, 78.1% had a family history of hypertension, and among those aged 36 to 45 years, 11.4% were in the first gestation, and 26.3% in the second gestation. Considering the associated conditions, diabetes prevailed with 50%; obesity with 22.2%, and the most selected foods for consumption among pregnant women, 47.5% had high energy content (processed/ultraprocessed).

**Conclusion:**

After an epidemiological analysis of the prevalence of hypertension, pregnant women with chronic hypertension, preexisting hypertension diagnosed during pregnancy, and hypertensive disease of pregnancy were identified. Regarding the possible factors associated with arterial hypertension, higher age, family history of hypertension, preexistence of hypertension, late pregnancies, diabetes, obesity and frequent consumption of processed/ultraprocessed foods were found.

## INTRODUCTION

Hypertension (HTN) is a clinical condition characterized by persistent levels of systolic (≥140mmHg) and diastolic (≥90mmHg) blood pressure, confirmed by two measures made on the upper right limb with the patient sitting at rest, with 4 to 6-hour intervals, for a minimal period of 2 weeks.^( 1 , 2 )^

Classified as primary (essential) or secondary, it differs according to its origin, where primary is idiopathic, and secondary derives from other pathologies, such as diabetes, obesity, and dyslipidemia. Both require careful laboratory control, treatment with medications, and, in certain cases, even surgery.^( 1 , 3 )^

A condition with high incidence and mortality rates, HTN prevails in almost half of the Brazilian population and progresses worldwide to death, being classified as a longitudinal progressive pandemic.^( 1 , 4 )^

It is evident that, besides its high mortality rate, it also predisposes towards the development of other cardiovascular and renal diseases, such as stroke, acute myocardial infarction, and renal failure.^( 1 , 4 )^

The diagnosis of HTN is obtained from routine assessments of blood pressure levels, sometimes supplemented by specific laboratory tests, clinical and epidemiologic analyses, but frequently the first intervention is late, since the patient is not always symptomatic.^( 1 , 5 )^ Consequently, when the characteristic symptoms appear, there already may be systemic complications and even lesions in target organs, such as the brain, heart, lungs, and kidneys. If not diagnosed and treated early, the morbidity and mortality risks are higher.^( 1 , 5 )^

The main epidemiological risk factors of HTN are excessive ingestion of sodium, family history, ethnicity, diabetes, obesity, hypothyroidism, tension, alcohol ingestion, unbalanced diet, sedentarism, psychological factors, dyslipidemia, smoking, and socioeconomic, socioenvironmental, and cultural factors.^( 1 , 4 , 5 )^

In pregnant women, the prevalence of HTN is equally high, considering the preexistent cases and those who develop the condition throughout pregnancy. With a high percentage of incidence in Brazil and the world, HTN manifests in pregnant women of all ages and is the major cause of maternal death in obstetrics.^( 2 , 6 - 9 )^ Studies conducted in Helsinki, Finland, and in Greece, revealed that children of mothers who are currently affected by complications related to HTN during gestation, in the future may present with cognitive deficiency, psychiatric problems, and a greater tendency to present with metabolic syndrome.^( 2 , 9 )^ As an important public health and women’s health problem, its incidence is higher in first-pregnant, multi-pregnant with advanced age for pregnancy, and obese pregnant women with family history of high blood pressure. The pregnant women in this condition also present with a greater risk of developing gestational diabetes and type 2 diabetes.^( 8 , 10 , 11 )^

Preexistent hypertension in pregnancy can be diagnosed before the conception or up to the 20^th^ week, and after this date and up to 42 days post-partum, it is considered hypertensive disease of pregnancy (HDP).^( 2 , 4 , 9 )^ The data exposed here clarifies the reasons for which the disease is characterized as a high-risk situation for pregnancy.^( 6 , 8 )^

The Ministry of Health regards high-risk pregnancy as the condition in which the life or health of the mother or of the fetus/newborn are at risk.^( 12 , 13 )^

Complications of hypertension in pregnancy are mainly miscarriage, premature delivery, restriction of fetal growth, detachment of the placenta, fetal distress, and diseases in vital organs after birth.^( 8 , 14 - 16 )^ The most severe situation, however, is when the disease progresses to preeclampsia, eclampsia, or hemolysis, elevated liver enzymes and low platelets (HELLP) syndrome, which are high-risk syndromes for maternal life.^( 17 - 19 )^ In this way, it is important to investigate which epidemiologic factors contribute to the appearance of HTN in pregnant women.

## OBJECTIVE

To investigate the epidemiological data of hypertension in pregnant women, as well as to identify its possible associated events.

## METHODS

This is a descriptive study of prevalence. The age defined for the study was 10 to 55 years, as per the epidemiological bulletin of the Ministry of Health,^( 13 )^ which points to pregnant women as of 10 years of age, having found, however, pregnant women aged between 18 and 45 years. Data collection was performed at the *Hospital do Servidor Público Estadual* (HSPE) *“Francisco Morato de Oliveira”,* a public hospital located in São Paulo (SP), the gynecology outpatient clinic (high risk prenatal) and the maternity ward in the morning and afternoon, from October 2015 to July 2016. A questionnaire with 22 questions prepared by the researchers, presented and approved by the Ethics Committee was applied. The sample of 114 women was based on the average monthly attendance of consultations of high-risk pregnant women. All appointments were scheduled through the Internet: www.iamspe.sp.gov.br, by telephone at (011) 55837001 and by the face-to-face service located at the call center Ibirapuera Avenue, 981 - São Paulo (SP) | Zip code: 04029-000 | CNPJ: 60,747,318 / 0001-62. Therefore, all information recorded in the MV Gynecology System database (source: IASMPE - HSPE Gynecology Directorate). The margin of error was 5%, and the confidence level, 95%. For the calculation, the equality of two proportions test, Pearson’s correlation, and analysis of variance (ANOVA) were used.

The pregnant women diagnosed with HTN were included in the research. When the investigation was finalized, with statistical assistance, data was tabulated in the (SPSS), version 20; Minitab 16; and Excel Office 2010 software.

All pregnant women read and signed the Informed Consent Form. This document complied with the regulations of Resolution 466/12, of the National Health Council, registered at *Plataforma* Brazil, and submitted to the Ethics Committee of the *Instituto de Assistência Médica ao Servidor Público Estadual* (IAMSPE) with favorable opinion N^o^ 1.197.330, CAAE: 47993315.4.00005.463.

## RESULTS

The sample studied was composed of 114 pregnant women seen at the outpatients clinic and maternity wards, during the period from October 2015 to July 2016. The subjects were civil servants or their dependents, who had a right to the services rendered by the HSPE. The pregnant women who performed external activities had their functions in the areas of operational, medium, technical and superior levels.

They received outpatient care, and 26 (22.8%) were hospitalized to control hypertension and other complications. Some women were admitted for one to 3 hospitalizations. By the conclusion of this investigation, 24 (21.1%) reached the end of pregnancy with the birth of their children. There were no emergency/urgency reports that resulted in death of the mother or the child.

Table 1 shows 71 (62.3%) pregnant women aged 18 to 35 years, 70 (61.4%) were white, 79 (69.3%) were married, and 12 (10.5%) were on a stable relationship (common-law marriage); 32 (28.1%) aged 18-35 years were in their first pregnancy and 39 (34.2%) in their second pregnancy; 91 (79.8%) women resided in their own homes, 75 (65.8%) lived with up to 5 people in the same house; 95 (83.3%) were employed; 53 (46.5%) received 1 to 3 minimum wages (minimum monthly wage of R$ 880.00, in 2016), and 43 (37.7%) had completed university courses.

**Table 1:** Sociodemographic data of hypertensive pregnant women (n=114)

	n (%)	
Age group, years		
18-35	71 (62.3)	
36-45	43 (37.7)	
46-55	0 (0)	
Skin color		
White	70 (61.4)	
Black	13 (11.4)	
Brown	31 (27.2)	
Marital status		
Married	79 (69.3)	
Single	22 (19.3)	
Common law marriage	12 (10.5)	
Widow	1 (0.9)	
Number of gestations	First	Second or more
18-35 years	32 (28.1)	39 (34.2)
36-45 years	13 (11.4)	30 (26.3)
46-55 years	0 (0)	0 (0)
Own home		
Yes	91 (79.8)	
No	23 (20.2)	
Residing in the same home		
1-5 people	75 (65.8)	
More than 6 people	33 (28.9)	
Lives alone	6 (5.3)	
Work conditions (employed)		
Yes	95 (83.3)	
No	19 (16.7)	
Family income, minimum wages*		
1-3	53 (46.5)	
3-5	39 (34.2)	
Over 5	13 (11.4)	
No answer	9 (7.9)	
Schooling level		
Incomplete junior school	9 (7.9)	
Complete junior school	5 (4.4)	
Incomplete senior school	8 (7.0)	
Complete senior school	24 (21.1)	
Incomplete university education	21 (18.4)	
Complete university education	43 (37.7)	
Graduate education	4 (3.5)	

* The minimum wage at the time of the study was R$ 880.00.

Table 2 shows that 49 (43%) pregnant women had chronic hypertension; 38 (33.3%) presented with HTN up to 20 weeks of pregnancy, and 27 (23.7%) more than 20 weeks of pregnancy; 36 (31.6%) had presented with HTN in other gestations. In the present study, except for the first-pregnant (45 pregnant women), with the remaining (69 pregnant women) in previous pregnancies, 14 (20.2%) had abortions, 19 (27.4%) had cesarean births and 36 (52.4%) had normal deliveries. When the disease was diagnosed, 114 (100%) pregnant women were medicated and maintained drug therapy; 89 (78.1%) had family history of HTN, 114 (100%) reported not consuming alcoholic beverages or smoking, and 68 (59.6%) initiated sexual activity at an age between 15 and 19 years. Seventy-eight (68.4%) pregnant women had no comorbidities, and, when present half of them accounted for diabetes. Of the foods most often chosen for consumption, 55 (47.5%) were in the group of processed/ultraprocessed foods. To relate such condition, the foods chosen were included in the analysis.

**Table 2:** Clinical/obstetric data of hypertensive pregnant women (n=114)

Clinical and obstetric data	n (%)
Hypertension before pregnancy	
Yes	49 (43)
No	65 (57)
Weeks of gestation when hypertension was diagnosed	
Up to 20 weeks	38 (33.3)
After 20 weeks	27 (23.7)
They were already hypertensive	49 (43.0)
For hypertensive women – had hypertension in other gestations?	
Yes	36 (31.6)
No	78 (68.4)
Past obstetric history n=69	
Miscarriage	14 (20.2)
Vaginal delivery	36 (52.4)
Cesarean section	19 (27.4)
Was medicated upon identification of hypertension?	
Yes	114 (100)
No	0 (0)
Still on treatment for hypertension	
Yes	114 (100)
No	0 (0)
Family history of hypertension	
Yes	89 (78.1)
No	25 (21.9)
Consumption of alcohol	
Yes	0 (0)
No	114 (100)
Smoking	
Yes	0 (0)
No	114 (100)
Age at initiating sexual activity	
15-19 years	68 (59.6)
Above 19 years	46 (40.4)
Association with other diseases	
Yes	36 (31.6)
No	78 (68.4)
Associated diseases	
Diabetes	18 (50)
Asthma	2 (5.6)
Hypothyroidism	5 (13.8)
Obesity	8 (22.2)
Depression	1 (2.8)
Arrhythmia	1 (2.8)
Labyrinthitis	1 (2.8)
Types of foods consumed	
Building (protein)	37 (32.6)
Energy (processed/ultraprocessed)	55 (47.5)
Regulating (fruits and green vegetables)	22 (19.9)

## DISCUSSION

The epidemiological profile of hypertensive pregnant women analyzed is very close to that presented in studies carried out in Brazil and in other countries.^( 4 , 8 , 9 , 15 - 17 )^

The results showed older pregnant women compared to those of other studies,^( 4 , 7 , 8 , 18 )^ since 62.3% of pregnant women were aged 18 to 35 years, and 37.7% were 36 to 45 years. Such figures point to late gestations, corroborating information from the Ministry of Health that highlights these ages as maternal risk factors.^( 13 )^ Data obtained by the *Instituto da Mulher* , in the city of Francisco Beltrão (PR), demonstrated a higher rate: 82% of pregnant women were aged 15 to 35 years. This total included ages between 15 and 17 years, a fact that did not occur in this present study.^( 18 )^

As to ethnicities, this research presented 61.4% of white women, different from other studies searched,^( 1 , 9 )^which presented black as prevalent for hypertension. It is important to point out that such a characteristic may be related to the profiles of pregnant women and the location where the study was conducted. Results similar to those of this investigation were found in another study of pregnant women with hypertensive syndromes, with 62.3% of white subjects.^( 18 )^

The results for this population show that the majority of women prefer to get pregnant within a more consolidated relationship, since stability coloborates for the evolution of pregnancy, of emotional status, and in terms of finances.^( 8 )^ Still, 69.3% reported being married and 10.5% were in a stable relationship. These data are similar to those of the study by Morais et al., which recorded 71.05% of pregnant women with hypertension were married and 15.79% were in a stable relationship.^( 17 )^

As to number of gestations, the rates of older women were confirmed. This is because in the age group 36 to 45 years, 13 (11.4%) women were still in their first pregnancy, and 30 (26.3%) in the second pregnancy or more, characterizing the later age for these pregnant women.^( 17 )^ It was noted that 37.7% of pregnant women had completed university education, a fact that may partly justify the older age. A higher rate (50% of those women had completed university education) was found in a study from the *Centro de Saúde da Região Central do Porto* , in Portugal, with immigrant and Portuguese pregnant women.^( 19 )^

Of the total, 65.8% of pregnant women resided with one to five other people, and 28.9% lived with more than five people. Oliveira et al.,^( 16 )^ presented higher rates in a study of hypertensive pregnant women, with 27.5% of them reporting living with up to five family members, and the other 72.5% living with more than five people in the same house. They also showed that the increase in number of “housemates” is proportional to the gestational risk. It generates tension, complications in pregnancy, discomfort, and greater financial expenses, and family conflicts.^( 16 )^

In this study, 83.3% of pregnant women worked outside the home. Women with external activities are generally more well informed, they exchange information relating to other people, and develop unique attitudes regarding pregnancy care.^( 4 )^ At the *Instituto da Mulher,* Francisco Beltrão (PR), 63.7% had jobs.^( 18 )^

As to family monthly income, 46.5% received from one to three minimum wages, 34.2% from three to five minimum wages, and 11.4% more than five minimum wages. In a global analysis, one can conclude that the socioeconomic level of the women in the study was, on average, satisfactory, allowing purchase of their own home, better transportation conditions to medical visits, and health-related care during pregnancy. This fact was confirmed in a research study in a public maternity in Teresina (PI), where a lower purchasing power was described, from one to three minimum wages in 45.4% of pregnant women with hypertension.^( 4 )^ As to the age the interviewees initiated sexual activity, 59.6% were at 15 to 19 years. A project carried out in a reference public school in the city of Bezerros (PE) showed that 59.3% of adolescents started their sexual activity between 15 and 19 years of age.^( 20 )^

In the present study, except for the first-pregnant (45 pregnant women), with the remaining (69 pregnant women) in previous pregnancies, 20.2% had abortion and 27.4% had cesarean deliveries, considering that for some pregnant women more than an event. At the primary care unit of the city of Paranavaí (PR), 26.8% of pregnant women reported one to three previous miscarriages. Both studies suggested that HTN is a risk factor for miscarriages.^( 8 )^ As to caesarian deliveries, the prevalence in Brazil is approximately 56%. The justification for this rate has not yet been fully defined, and HTN is not mentioned as a determining factor for this procedure.^( 8 - 12 , 13 )^ The Ministry of Health recommends vaginal deliveries for high-risk pregnant women.^( 8 - 12 , 13 )^

Regarding gestational age, it is more common that HTN be diagnosed as of the 20^th^ week, a fact that defines HDP,^( 2 , 6 )^ and in this study, it was observed in 23.7% of pregnant women. In 33.3% of them, hypertension occurred with up to 20 weeks of pregnancy, revealing unknown preexisting hypertension. Such a fact is based on studies conducted in the states of Brazil and of other countries that consider, in these cases, preexisting hypertension.^( 2 , 4 , 9 )^It is important to point out that among the pregnant women studied, 43% had a declared diagnosis of chronic hypertension before the current pregnancy. Thus, almost half of the pregnant women of this study became pregnant while already being hypertensive, which highlighted the importance of prenatal care beginning early.^( 6 , 21 )^

Considering antihypertensive medications, 100% of pregnant women were medicated when diagnosed with hypertension and continuously complied with treatment. This shows the commitment of the population studied to the treatment. In the study carried out by Morais et al.,^( 17 )^ the following classifications were considered: high compliance (total compliance) in 18.4%, medium compliance in 63.2%, low compliance in 18.4%. The factors that most collaborated with the classification of these rates were forgetfulness and the onset of unpleasant side effects when the medications were taken, factors that decreased the frequency of daily use of antihypertensive medications.^( 17 )^ In both studies, the local socioeconomic development of the sites, the level of instructions, the local conditions, and the cultural aspects of the populations studied need to be considered.

Of the total number of respondents, 78.1% stated they had a family history of hypertension, and this finding was the clinical datum of highest incidence in the current research. In a study at the *Hospital Universitário de Maceió* in the city of Alagoas (AL), 54.6% of pregnant women with hypertension had a family history of the disease, and the incidence was higher in women with closer kin affected by HTN (father and mother).^( 16 )^ This highlights this condition as important factor for classifying the pregnancy as high-risk.^( 16 )^

As to consumption of alcohol and tobacco, 100% of pregnant women stated not having such habits. The results of this study reflect in the study population’s profile, which showed awareness of the risks that occurred in using such substances, since most of them worked in the areas of healthcare, education, and administration, and were in constant exchanges of knowledge with other people. Yet on the topic of ingestion of alcoholic beverages and use of tobacco, the results suggested controversy, such as the evasion of information, perhaps due to embarrassment in the presence of the investigator, or to feeling responsible for possible future damage to their child. Rocha et al., found a different rate: 11.3% were smokers and 16% consumed alcoholic beverages.^( 22 )^

It is important to point out that HTN is associated with other diseases. We found 31.6% of association in pregnant women, in which diabetes was the comorbidity in 50% of cases. Diabetes was also associated with obesity and higher age, two important risk factors. These data were confirmed in other studies on diabetes.^( 23 - 25 )^

During the analysis of the associations, another condition was discovered: of the total self-reported conditions, 22.2% were obesity. A study at the *Hospital Universitário de Maceió* in the city of Alagoas (AL), concluded that the body mass index aggravated by pregnancy contributed to the onset of diabetes, hypertension, high rate of elective surgical or emergency deliveries, postpartum hemorrhage, as well as the permanence of obesity after birth.^( 16 )^

The choice of foods that were predominantly eaten during pregnancy resulted in 47.5% of processed/ultraprocessed food (soft drinks, sweets, industrialized snacks, pasta, cold meats, and artificial seasonings).^( 26 , 27 )^Some studies have demonstrated that inadequate nutrition can be associated with the onset of hypertension, diabetes, overweight, and obesity, bringing with it other risks and damages to the body, and making treatment difficult.^( 26 , 27 )^ The care delivered to the pregnant women of this study played a crucial role in the prevention of maternal and perinatal morbidity and mortality, since preeclampsia in pregnancy still cannot be impeded in all cases, but in most cases, maternal death can be prevented.

## CONCLUSION

After epidemiologic analysis of the results of hypertension, pregnant women were found with chronic hypertension, preexistent hypertension diagnosed during gestation, and hypertensive disease of pregnancy. As to the possible factors associated with hypertension, the following were identified: older pregnant women, family history of hypertension, preexisting hypertension, late pregnancies, diabetes, obesity, and frequent consumption of processed/ultraprocessed food.
