# Supplementary material for: Epidemiology of artherial hypertension in pregnants
Source: Einstein (Sao Paulo). 2019 Oct 22;18:eAO4682. doi: 10.31744/einstein_journal/2020AO4682 (PMC6896657; doi:10.31744/einstein_journal/2020AO4682)
Supplement: Translation

# Translation: Epidemiologia da hipertensão arterial em gestantes

**Authors:** Marilda Gonçalves de Sousa, Reginaldo Guedes Coelho Lopes, Maria Luiza Toledo Leite Ferreira da Rocha, Umberto Gazi Lippi, Edgar de Sousa Costa, Célia Maria Pinheiro dos Santos

**Affiliations:** 1 Brasil Instituto de Assistência Médica ao Servidor Público Estadual, São Paulo, SP, Brasil.; 2 Brasil Universidade Federal de São Paulo, São Paulo, SP, Brasil.

**Keywords:** Complicações na gravidez/epidemiologia, Hipertensão induzida pela gravidez/epidemiologia, Hospitais, Serviços de Saúde

## Abstract

**Objetivo:**

Pesquisar os dados epidemiológicos da hipertensão arterial em gestantes, bem como identificar seus possíveis eventos associados.

**Métodos:**

A coleta de dados foi realizada no ambulatório do pré-natal de alto risco e na enfermaria da maternidade em hospital público da cidade de São Paulo, nos períodos matutino e vespertino, de outubro de 2015 a julho de 2016. Foi aplicado um questionário com 22 perguntas elaborado pelos pesquisadores. A margem de erro foi de 5% e o nível de confiança, de 95%. Para o cálculo, foram usados o teste de igualdade de duas proporções, a correlação de Pearson e o teste de ANOVA.

**Resultados:**

Dentre as entrevistadas, 43% tinham hipertensão crônica, 33,3% se apresentaram com até 20 semanas de gestação, 23,7% se apresentaram após a 20ª semana da gestação, 62,3% tinham idade entre 18 e 35 anos, 78,1% tinham antecedente familiar com hipertensão arterial, 11,4% com idade entre 36 a 45 anos estavam na primeira gestação, e 26,3% com a mesma idade estavam a partir da segunda gestação. Dentre as afecções associadas, prevaleceu o diabetes com 50%; 22,2% se tratavam de obesidade, e dos alimentos mais escolhidos para consumo entre as gestantes, 47,5% possuíam alto teor energético (processados/ultraprocessados).

**Conclusão:**

Após análise epidemiológica no resultado da prevalência da hipertensão arterial, foram encontradas gestantes com hipertensão arterial crônica, hipertensão arterial preexistente descoberta durante a gestação e doença hipertensiva específica da gestação. Em relação aos possíveis fatores associados à hipertensão arterial, foram encontrados: idade mais elevada, antecedentes familiares de hipertensão, preexistência de hipertensão, gestações tardias, diabetes, obesidade e frequente consumo de alimentos processados/ultraprocessados.

## INTRODUÇÃO

A hipertensão arterial sistêmica (HAS) é uma condição clínica caracterizada por níveis persistentes de pressão arterial sistólica (≥140mmHg) e diastólica (≥90mmHg), confirmadas em duas aferições no membro superior direito com o paciente em repouso sentado, em intervalos de 4 a 6 horas, por um período mínimo de 2 semanas.^( 1 , 2 )^

Classificada como primária (essencial) ou secundária, diferencia-se segundo a origem, sendo a primária idiopática, e a secundária, quando deriva de outras patologias, como diabetes, obesidade e dislipidemia. Ambas têm necessidade de controle laboratorial cuidadoso, tratamento com medicamentos e, em certos casos, até cirúrgico.^( 1 , 3 )^

Afecção de elevada incidência e mortalidade, a HAS prevalece em quase metade da população brasileira e evolui mundialmente em mortalidade, sendo classificada como pandemia longitudinalmente progressiva.^( 1 , 4 )^

Evidencia-se que, além de sua alta mortalidade, também predispõe ao desenvolvimento de outras doenças cardiovasculares e renais, como acidentes vasculares cerebrais, infarto agudo do miocárdio e falência renal.^( 1 , 4 )^

O diagnóstico da HAS é obtido de aferições rotineiras dos níveis pressóricos, por vezes complementadas por exames laboratoriais específicos, análises clínicas e epidemiológicas, mas a primeira intervenção frequentemente é tardia, pois nem sempre o paciente é sintomático.^( 1 , 5 )^Consequentemente, quando surgem os sintomas característicos, já pode existir complicações sistêmicas e até mesmo lesões em órgãos-alvo, como encéfalo, coração, pulmões e rins. Se não diagnosticada e tratada precocemente, o risco de morbimortalidade é maior.^( 1 , 5 )^

Os principais fatores de riscos epidemiológicos de HAS são consumo excessivo de sódio, antecedentes familiares, de etnia, diabetes, obesidade, hipotireoidismo, tensão, ingestão de álcool, dieta alimentar desregrada, sedentarismo, fatores psicológicos, dislipidemia, tabagismo e fatores socioeconômicos, socioambientais e culturais.^( 1 , 4 , 5 )^

Quando em gestantes, a prevalência da HAS é igualmente elevada, considerando as preexistentes e aquelas que desenvolvem a afecção no decorrer da gestação. Com alto percentual de incidência no Brasil e no mundo, a HAS manifesta-se em gestantes de todas as idades e é a maior causa de morte materna em obstetrícia.^( 2 , 6 - 9 )^Estudos realizados na cidade de Helsinquia, na Finlândia, e na Grécia revelam que filhos de mães que são atualmente acometidas por complicações relacionadas à HAS na gestação, podem futuramente apresentar deficiência cognitiva, problemas psiquiátricos e maior tendência a sofrer síndrome metabólica.^( 2 , 9 )^Problema importante de saúde pública e saúde da mulher incide mais comumente em primigestas, multíparas com idade tardia para a gravidez, gestante obesa e naquelas com antecedente familiar de hipertensão arterial. As gravidas nestas condições também apresentam maior risco de desenvolver diabetes gestacional e diabetes tipo 2.^( 8 , 10 , 11 )^

A hipertensão preexistente na gravidez pode ser diagnosticada antes da concepção ou até a 20ª semana, sendo considerada doença hipertensiva específica da gestação (DHEG) após esta data e até 42 dias pós-parto.^( 2 , 4 , 9 )^Os dados aqui expostos esclarecem os motivos pelos quais a afecção é caracterizada como situação de alto risco para a gravidez.^( 6 , 8 )^

O Ministério da Saúde conceitua gestação de alto risco a condição na qual a vida ou a saúde da mãe ou do feto/recém-nascido correm risco.^( 12 , 13 )^

As complicações da hipertensão na gestação são principalmente abortamento, parto prematuro, restrição do crescimento fetal, descolamento da placenta, sofrimento fetal e afecções em órgãos vitais após o nascimento.^( 8 , 14 - 16 )^A situação mais grave, no entanto, é quando a doença evolui para pré-eclâmpsia, eclâmpsia ou síndrome hemólise, elevação de enzimas hepáticas e baixa contagem de plaquetas (HELLP), que são síndromes de elevado risco para a vida materna.^( 17 - 19 )^Desta forma é importante pesquisar quais fatores epidemiológicos contribuem para o surgimento da HAS em gestantes.

## OBJETIVO

Pesquisar os dados epidemiológicos da hipertensão arterial em gestantes, bem como identificar seus possíveis eventos associados.

## MÉTODOS

Estudo descritivo de prevalência. A idade definida para o estudo foi de 10 a 55 anos, conforme boletim epidemiológico do Ministério da Saúde,^( 13 )^que aponta mulheres grávidas a partir dos 10 anos, tendo sido encontradas, porém, gestantes com idade entre 18 a 45 anos. A coleta de dados foi realizada no Hospital do Servidor Público Estadual (HSPE) “Francisco Morato de Oliveira”, um hospital público localizado em São Paulo (SP), no ambulatório de ginecologia (pré-natal de alto risco) e na enfermaria da maternidade, nos períodos matutino e vespertino, de outubro de 2015 a julho de 2016. Foi aplicado um questionário com 22 perguntas elaborado pelos pesquisadores, apresentado e aprovado pelo Comitê de Ética. A amostra de 114 mulheres foi fundamentada pela média mensal do atendimento das consultas das gestantes de alto risco. Todas as consultas foram agendadas pela Internet: www.iamspe.sp.gov.br, pelo telefone de número (011) 55837001 e pelo atendimento presencial localizado na central de atendimento Avenida Ibirapuera, 981 – São Paulo (SP) | CEP: 04029-000 | CNPJ: 60.747.318/0001-62. Ficando, portanto todas as informações registradas no banco de dados MV sistema de consulta do ambulatório de ginecologia, (fonte: Diretoria de Ginecologia do IASMPE – HSPE). A margem de erro foi de 5%, e o nível de confiança, de 95%. Para o cálculo, foram usados o teste de igualdade de duas proporções, a correlação de Pearson e o teste de Análise de Variância (ANOVA).

Foram incluídas na pesquisa gestantes com diagnóstico de HAS. Finalizada a pesquisa, com auxilio estatístico, foram tabulados os dados nos softwares (SPSS), versão 20; Minitab 16; e Excel Office 2010.

Todas gestantes leram e assinaram o Termo de Consentimento Livre e Esclarecido (TCLE). Foram atendidas as normas da resolução 466/12, do Conselho Nacional de Saúde, cadastrado na Plataforma Brasil e submetido ao Comitê de Ética do Instituto de Assistência Médica ao Servidor Público Estadual (IAMSPE) com parecer favorável 1.197.330, CAAE: 47993315.4.00005.463.

## RESULTADOS

A amostra estudada foi de 114 gestantes atendidas no ambulatório e nas enfermarias da maternidade, no período de outubro de 2015 a julho de 2016. Eram funcionárias públicas ou dependentes de funcionários que tinham direito de usufruir dos serviços prestados pelo HSPE. As gestantes que desempenhavam atividades externas tinham suas funções nas áreas de níveis operacionais, médio, técnico e superior. Receberam assistência ambulatorial, e 26 (22,8%) foram internadas para o controle da hipertensão arterial e outras intercorrências. Para algumas mulheres aconteceram de 1 a 3 internações. Até o término desta pesquisa 24 (21,1%) chegaram ao final da gestação com o nascimento dos seus filhos. Não houve relatos de emergência/urgência que resultassem em óbito de mãe ou filho.

Na tabela 1 , foram encontradas 71 (62,3%) gestantes com idade entre 18 a 35 anos, 70 (61,4%) eram da cor branca, 79 (69,3%) eram casadas e 12 (10,5%) viviam em união estável; 32 (28,1%) com idade entre 18 a 35 anos estavam na primeira gestação e 39 (34,2%) com a mesma idade na segunda gestação; 91 (79,8%) mulheres residiam em casa própria, 75 (65,8%) moravam com até 5 pessoas na mesma casa; 95 (83,3%) mantinham vínculo empregatício, 53 (46,5%) recebiam de 1 a 3 salários mínimos (salário mínimo de R$ 880,00, em 2016), e 43 (37,7%) possuíam Ensino Superior completo.

**Tabela 1:** Dados sociodemográficos das gestantes hipertensas (n=114)

	n (%)	
Faixa etária, anos		
18-35	71 (62,3)	
36-45	43 (37,7)	
46-55	0 (0)	
Cor da pele		
Branca	70 (61,4)	
Negra	13 (11,4)	
Parda	31 (27,2)	
Situação conjugal		
Casada	79 (69,3)	
Solteira	22 (19,3)	
União estável	12 (10,5)	
Viúva	1 (0,9)	
Quantidade de gestações	Primeira	Segunda ou mais
18-35 anos	32 (28,1)	39 (34,2)
36-45 anos	13 (11,4)	30 (26,3)
46-55 anos	0 (0)	0 (0)
Casa própria		
Sim	91 (79,8)	
Não	23 (20,2)	
Residem na mesma casa		
1-5 pessoas	75 (65,8)	
Mais de 6 pessoas	33 (28,9)	
Mora sozinha	6 (5,3)	
Condição ocupacional (empregada))		
Sim	95 (83,3)	
Não	19 (16,7)	
Renda familiar, salários mínimos*		
1-3	53 (46,5)	
3-5	39 (34,2)	
Acima de 5	13 (11,4)	
Não responderam	9 (7,9)	
Nível de escolaridade		
Fundamental incompleto	9 (7,9)	
Fundamental completo	5 (4,4)	
Médio incompleto	8 (7,0)	
Médio completo	24 (21,1)	
Superior incompleto	21 (18,4)	
Superior completo	43 (37,7)	
Pós-graduação	4 (3,5)	

* O salário mínimo no momento da pesquisa era de R$ 880,00.

A tabela 2 mostra que 49 (43%) gestantes tinham hipertensão crônica; 38 (33,3%) apresentaram HAS com até 20 semanas de gestação e 27 (23,7%) com mais de 20 semanas de gestação; 36 (31,6%) tinham apresentado HAS em outras gestações. No presente estudo, com exceção das primigestas (45 gestantes), com as demais (69 gestantes) em gestações anteriores 14 (20,2%) delas tiveram abortamento, 19 (27,4%) tiveram partos por cesárea e 36 (52,4%) tiveram partos normais. Ao ser diagnosticada a doença, 114 (100%) gestantes foram medicadas e mantiveram a terapia medicamentosa; 89 (78,1%) tinham antecedente familiar de HAS, 114 (100%) referiram não consumir bebida alcoólica ou tabaco, e 68 (59,6%) iniciaram atividade sexual com idade entre 15 e 19 anos. Setenta e oito (68,4%) gestantes não tinham comorbidades, e, quando presentes, metade eram casos de diabetes. Dos alimentos mais escolhidos para consumo, 55 (47,5%) faziam parte do grupo de alimentos processados/ultraprocessados. Para relacionar tal condição, foram tabulados os alimentos escolhidos.

**Tabela 2:** Dados clínicos/obstétricos das gestantes hipertensas (n=114)

Dados clínicos e obstétricos	n (%)
Hipertensão antes da gravidez	
Sim	49 (43)
Não	65 (57)
Semanas de gravidez na qual a hipertensão foi diagnosticada	
Até 20ª semanas	38 (33,3)
Após 20ª semanas	27 (23,7)
Eram hipertensas	49 (43,0)
Para as hipertensas, tiveram hipertensão em outras gestações	
Sim	36 (31,6)
Não	78 (68,4)
Antecedentes obstétricos n=69	
Abortamento	14 (20,2)
Parto normal	36 (52,4)
Parto cesárea	19 (27,4)
Ao identificar a hipertensão, foi medicada	
Sim	114 (100)
Não	0 (0)
Ainda faz tratamento para hipertensão	
Sim	114 (100)
Não	0 (0)
Antecedente familiar de hipertensão	
Sim	89 (78,1)
Não	25 (21,9)
Consumo de bebida alcoólica	
Sim	0 (0)
Não	114 (100)
Tabagismo	
Sim	0 (0)
Não	114 (100)
Idade de início da atividade sexual	
15-19 anos	68 (59,6)
Acima de 19 anos	46 (40,4)
Associação com outras afecções	
Sim	36 (31,6)
Não	78 (68,4)
Afecções associadas	
Diabetes	18 (50)
Asma	2 (5,6)
Hipotireoidismo	5 (13,8)
Obesidade	8 (22,2)
Depressão	1 (2,8)
Arritmia	1 (2,8)
Labirintite	1 (2,8)
Tipos de alimento consumidos	
Construtores (proteína)	37 (32,6)
Energéticos (processado/ultraprocessado)	55 (47,5)
Reguladores (fruta e hortaliça)	22 (19,9)

## DISCUSSÃO

O perfil epidemiológico das gestantes hipertensas encontradas se aproxima do apresentado em estudos realizados no Brasil e em outros países.^( 4 , 8 , 9 , 15 - 17 )^

Os resultados evidenciaram gestantes mais velhas comparadas às de outros estudos,^( 4 , 7 , 8 , 18 )^já que 62,3% das gestantes tinham entre 18 e 35 anos, e 37,7% tinham entre 36 a 45 anos. Tais números apontaram para gestações tardias corroborando informações do Ministério da Saúde, que apontam estas idades como fator de riscos materno.^( 13 )^Dados obtidos pelo Instituto da Mulher, em Francisco Beltrão (PR), registraram índice superior: em 82% das gestantes, a idade variou de 15 e 35 anos. Este total incluiu idades entre 15 e 17 anos, o que não ocorreu no presente estudo.^( 18 )^

Quanto às etnias, esta pesquisa apresentou 61,4% das mulheres de cor branca, diferente de outros estudos consultados,^( 1 , 9 )^que apresentaram a cor negra como prevalente para hipertensão arterial. É importante ressaltar que tal característica pode estar relacionada aos perfis das gestantes e do local onde o estudo foi realizado. Resultado semelhante ao desta pesquisa foi encontrado em outro estudo de gestantes com síndromes hipertensivas, com 62,3% de cor branca.^( 18 )^

Os resultados para esta população deixam evidente que a maioria das mulheres prefere engravidar dentro de um relacionamento mais consolidado, uma vez que a estabilidade colabora para a evolução da gestação, do estado emocional e financeiramente.^( 8 )^Ainda, 69,3% reportaram ser casadas e 10,5% tinham união estável. São dados aproximados aos de estudo de Morais et al., que registraram 71,05% das gestantes com hipertensão casadas e 15,79% em união consensual.^( 17 )^

Referente ao número de gestações, foi confirmado índice de mulheres com mais idade. Isso porque 13 (11,4%) com 36 a 45 anos ainda estavam na primeira gestação e 30 (26,3%) com a mesma idade estavam a partir da segunda gestação, caracterizando a idade tardia para estas gestantes.^( 17 )^Foi observado que 37,7% das gestantes tinham Ensino Superior completo, fato que pode justificar em parte o adiamento da maternidade. Índice maior (50% com Ensino Superior completo) foi encontrado em estudo no Centro de Saúde da Região Central do Porto, em Portugal, com gestantes imigrantes e portuguesas.^( 19 )^

Do total, 65,8% das gestantes residiam com uma a cinco pessoas, e 28,9% moravam com mais de cinco pessoas. Oliveira et al.,^( 16 )^apresentaram índices maiores em estudo de gestantes hipertensas, com 27,5% relatando residir com até cinco membros da família e as demais 72,5% morando com mais de cinco membros na mesma casa. Também evidenciaram que o aumento do número de companhias domiciliares é proporcional ao risco gestacional. Gera na gestante tensão, complicações na gravidez, desconforto, mais gastos financeiros e conflitos familiares.^( 16 )^

Neste estudo, 83,3% das gestantes trabalhavam fora de casa. Mulheres com suas ocupações externas em geral são mais esclarecidas, trocam informações relacionando-se com outras pessoas e constroem atitudes próprias referentes aos cuidados na gestação.^( 4 )^No Instituto da Mulher, Francisco Beltrão (PR), 63,7% trabalhavam em serviços externos.^( 18 )^

Quanto à renda familiar, 46,5% recebiam de um a três salários mínimos, 34,2% de três a cinco salários, e 11,4% acima de cinco salários mínimos. Em análise geral, pode-se concluir que o nível socioeconômico das pesquisadas foi, em média, satisfatório, permitindo aquisição de moradia, melhor condição do deslocamento para consultas e cuidados relacionados à sua saúde durante a gestação. Este fato foi confirmado em pesquisa em maternidade pública em Teresina (PI), onde foi encontrado menor poder aquisitivo, de um a três salários mínimos em 45,4% das gestantes com hipertensão.^( 4 )^Acerca do período em que as entrevistadas iniciaram a atividade sexual, 59,6% o fizeram com idade entre 15 e 19 anos. Trabalho desenvolvido em escola pública de referência em Bezerros (PE) mostrou que 59,3% das adolescentes tiveram sua primeira atividade sexual entre 15 e 19 anos.^( 20 )^

No presente estudo, com exceção das primigestas (45 gestantes), com as demais (69 gestantes) em gestações anteriores 20,2% delas tiveram abortamento e 27,4% tiveram partos por cesárea, tendo em vista que para algumas gestantes aconteceram mais de um evento. Na Unidade Básica de Saúde do Município de Paranavaí (PR), 26,8% das gestantes apresentaram anteriormente um a três episódios de abortamentos. Ambas as pesquisas sugerem que a HAS é fator de risco para o abortamento.^( 8 )^Em relação aos partos por cesárea, no Brasil, a prevalência está perto de 56%. A justificativa para este índice ainda não foi totalmente definida, e a HAS não é apontada como fator determinante para este procedimento.^( 8 - 13 )^O Ministério da Saúde aconselha o parto vaginal para gestante de alto risco.^( 8 - 13 )^

Referente à idade gestacional, é mais comum a HAS ser diagnosticada a partir da 20ª semana, fato que define a DHEG,^( 2 , 6 )^e que ocorreu nesta pesquisa com 23,7% das gestantes. Em 33,3% das grávidas a hipertensão arterial aconteceu com até 20 semanas de gravidez, revelando hipertensão prévia desconhecida. Tal fato tem como base estudos realizados nos Estados do Brasil e de outros países que referem nesses casos tratar-se de hipertensão preexistente.^( 2 , 4 , 9 )^Importante salientar que, dentre as gestantes estudadas, 43% tinham declarado o diagnóstico de hipertensão arterial crônica antes da atual gestação. Assim, quase metade das gestantes desta pesquisa ficou grávida já hipertensa, o que realçou a importância do pré-natal iniciar-se precocemente.^( 6 , 21 )^

Quanto à questão referente aos medicamentos anti-hipertensivos, 100% das gestantes foram medicadas diante do diagnóstico da hipertensão e aderiram continuamente ao tratamento. Isto mostra o comprometimento da população estudada com o tratamento. No estudo de Morais et al.,^( 17 )^foram consideradas as seguintes classificações: alta adesão (adesão total) em 18,4%; média adesão em 63,2%; baixa adesão em 18,4%. Os fatores que mais colaboraram com a variação da classificação desses índices foram o esquecimento e o surgimento de sinais e sintomas colaterais desagradáveis quando tomavam os medicamentos, fatores que as fizeram diminuir a frequência do uso diário dos medicamentos anti-hipertensivos.^( 17 )^Em ambos os estudos, devem ser considerados o desenvolvimento socioeconômico dos locais em questão, o nível de orientações, as condições do local de tratamento e os aspectos culturais das populações estudadas.

Do total de respondentes, 78,1% disseram ter antecedente familiar de hipertensão, e este achado foi o dado clínico mais incidente da presente pesquisa. Em estudo no Hospital Universitário de Maceió, Alagoas (AL), 54,6% das gestantes com hipertensão tinham histórico familiar da doença, e foi maior a incidência em mulheres cujo parentesco de HAS era mais próximo (pai e mãe).^( 16 )^Isto reforça que esta condição é fator importante para classificar a gestação como de alto risco.^( 16 )^

Quanto ao consumo de álcool e tabaco, 100% das gestantes disseram não cultivar tais hábitos. O resultado deste estudo reflete no perfil da população estudada, que mostrava ter ciência dos riscos que corriam quanto ao uso de tais substâncias, visto que a maioria trabalhava na área da saúde, da educação e da administração, e estava em constante troca de conhecimentos com outras pessoas. Ainda sobre a ingestão de bebida alcoólica e o uso de tabaco, os resultados sugeriram controvérsia, como a sonegação de informações, talvez pelo constrangimento diante da presença da pesquisadora ou por se sentirem responsáveis por eventual dano futuro ao filho. Rocha et al., encontraram índice diferente: 11,3% eram tabagistas e 16% consumiam bebida alcoólica.^( 22 )^

É importante salientar que a HAS se associa com outras doenças. Encontramos 31,6% de associações nas gestantes, sendo esta comorbidade o diabetes em 50% dos casos. O diabetes também se associa à obesidade e à idade tardia, dois importantes fatores de risco. Estes dados foram confirmados em outros estudos sobre diabetes.^( 23 - 25 )^

Durante a análise das associações, foi descoberta outra afecção: do total das afecções autodeclaradas 22,2% delas eram obesidade. Estudo no Hospital Universitário de Maceió, Alagoas (AL), concluiu que o índice de massa corporal agravado pela gestação contribui para o surgimento de diabetes, hipertensão arterial, alto índice de partos cirúrgicos eletivos ou por emergências, hemorragia pós-parto e, ainda, para a permanência da obesidade após o parto.^( 16 )^

A escolha dos alimentos que consumiram predominantemente durante a gestação resultou em 47,5% de alimentos processados/ultraprocessados (refrigerantes, doces, salgadinhos industrializados, massas, embutidos e temperos artificiais).^( 26 , 27 )^Estudos evidenciam que a nutrição inadequada pode estar associada ao surgimento da hipertensão arterial, diabetes, sobrepeso e obesidade, trazendo outros riscos e malefícios para o organismo, e dificultando o tratamento.^( 26 , 27 )^As assistências prestadas para as gestantes desta pesquisa exerceram papel de fundamental importância na prevenção da morbidade e das mortalidades materna e perinatal, pois a pré-eclâmpsia na gestação ainda não pode ser impedida em todos os casos, porém, o óbito materno, na maioria das ocasiões, pode ser prevenido.

## CONCLUSÃO

Após análise epidemiológica no resultado da prevalência da hipertensão arterial foram encontradas gestantes com hipertensão arterial crônica, hipertensão arterial preexistente descoberta durante a gestação e doença hipertensiva específica da gestação. Em relação aos possíveis fatores associados à hipertensão arterial, foram encontrados: gestantes com mais idades, antecedentes familiares de hipertensão, preexistência de hipertensão, gestações tardias, diabetes, obesidade e frequente consumo de alimentos processados/ultraprocessados.
